# Fidelity of implementation of national guidelines on malaria diagnosis for children under-five years in Rivers State, Nigeria

**DOI:** 10.1186/s12936-024-04957-4

**Published:** 2024-04-27

**Authors:** Mina Whyte, Latifat Ibisomi, Tobias Chirwa, Jonathan Levin, Wiedaad Slemming

**Affiliations:** 1 Division of Epidemiology and Biostatistics, School of Public Health, University of the Witwatersrand, Johannesburg, South Africa; 2 Department of Medicine, University of Otago, Wellington, New Zealand; 3 Nigerian Institute of Medical Research, Lagos, Nigeria; 4 Division of Community Paediatrics, Department of Paediatrics and Child Health, Faculty of Health Sciences, University of the Witwatersrand, Johannesburg, South Africa; 5 Children’s Institute, Department of Paediatrics and Child Health, Faculty of Health Sciences, University of Cape Town, Cape Town, South Africa

**Keywords:** Malaria, Implementation fidelity, Guidelines, Test and treat, Children, Nigeria

## Abstract

**Background:**

Malaria is still a disease of global public health importance and children under-five years of age are the most vulnerable to the disease. Nigeria adopted the “test and treat” strategy in the national malaria guidelines as one of the ways to control malaria transmission. The level of adherence to the guidelines is an important indicator for the success or failure of the country’s roadmap to malaria elimination by 2030. This study aimed to assess the fidelity of implementation of the national guidelines on malaria diagnosis for children under-five years and examine its associated moderating factors in health care facilities in Rivers State, Nigeria.

**Methods:**

This was a descriptive, cross-sectional study conducted in Port Harcourt metropolis. Data were collected from 147 public, formal private and informal private health care facilities. The study used a questionnaire developed based on Carroll’s Conceptual Framework for Implementation Fidelity. Frequency, mean and median scores for implementation fidelity and its associated factors were calculated. Associations between fidelity and the measured predictors were examined using Mann Whitney U test, Kruskal Wallis test, and multiple linear regression modelling using robust estimation of errors. Regression results are presented in adjusted coefficient (β) and 95% confidence intervals.

**Results:**

The median (IQR) score fidelity score for all participants was 65% (43.3, 85). Informal private facilities (proprietary patent medicine vendors) had the lowest fidelity scores (47%) compared to formal private (69%) and public health facilities (79%). Intervention complexity had a statistically significant inverse relationship to implementation fidelity (β = − 1.89 [− 3.42, − 0.34]). Increase in participant responsiveness (β = 8.57 [4.83, 12.32]) and the type of malaria test offered at the facility (e.g., RDT vs. no test, β = 16.90 [6.78, 27.03]; microscopy vs. no test, β = 21.88 [13.60, 30.16]) were positively associated with fidelity score.

**Conclusions:**

This study showed that core elements of the “test and treat” strategy, such as testing all suspected cases with approved diagnostic methods before treatment, are still not fully implemented by health facilities. There is a need for strategies to increase fidelity, especially in the informal private health sector, for malaria elimination programme outcomes to be achieved.

**Supplementary Information:**

The online version contains supplementary material available at 10.1186/s12936-024-04957-4.

## Background

Malaria is an infectious disease caused by *Plasmodium* parasites which are transmitted to humans through the bite of a female *Anopheles* mosquito [1]. Africa has the highest burden of the disease having an estimated 94% of global cases (233 million cases in 2022), with Nigeria, Democratic Republic of Congo, Uganda, and Mozambique accounting for about half of these cases [2]. In 2022, Nigeria was responsible for over a quarter (27%) of all malaria cases globally, with the highest percentage (31%) of all malaria deaths and has a high prevalence of *Plasmodium falciparum* parasites [2, 3]. Children under the age of five years are the most vulnerable to the disease. It is estimated that every minute a child aged under-five years dies from malaria, and in 2019, 20% of all deaths in this age group occurred in Nigeria [4]. Malaria also contributes to the poverty cycle through the reduction of productive labour time for adults, increase in missed school days for children, and greater health expenses for families and countries [5–8]. The significant morbidity and mortality caused by the disease, and its huge socioeconomic impact, made it a target of the Sustainable Development Goal 3 which aims to end the malaria epidemic, among other communicable diseases, by 2030 [9].

Substantial investments have been made towards achieving this Goal in malaria endemic regions by governments and developmental partners. There has been a scale-up of malaria intervention programmes like provision of insecticide treated nets, malaria rapid diagnostic tests (RDTs), and artemisinin-based combinations for treatment of uncomplicated malaria [2, 10, 11]. This contributed to a global downward trend in malaria morbidity and mortality between 2000 and 2015 [2], however, the gains seem to have stalled as malaria cases increased from an estimated 231 million in 2015 to 249 million in 2022 [2]. The emergence of drug-resistant parasites has been reported as one of the factors that contributes to the resurgence of malaria [12–14]. Amongst other factors, drug resitance is influenced by low drug levels when a newly acquired infection is exposed to waning anti-malarial levels from a previous treatment [15]. This can happen if an anti-malarial drug is taken when the individual does not have a malarial infection. The *Global Technical Strategy for Malaria 2016–2030* of the World Health Organization (WHO) discourages presumptive treatment of malaria and recommends universal access to diagnosis and treatment of malaria [1]. This is abbreviated as the “test and treat” strategy [16, 17].

In line with the WHO’s strategy, Nigeria developed the *National Guidelines for Diagnosis and Treatment of Malaria* (2015) to guide health care providers in performing parasitological diagnosis for all suspected cases of malaria [18]. It recommends microscopy tests for secondary and tertiary level health care facilities, while RDTs can be used at facilities at all levels, and in communities [18]. However, there have been challenges with adherence to the malaria “test and treat” strategy with adherence falling short of the target of 100% globally [19–22]. The situation is similar in Nigeria. For instance, the 2018 Nigeria Demographic and Health Survey, reported diagnosis before treatment to be at 11.8% nationally [23]. Two studies conducted in Southern Nigeria reported high incidences of presumptive case management, with only about one third of patients tested before treatment with anti-malarial drugs [24, 25]. Another study conducted in South-West Nigeria, among health care workers in public and private facilities, reported strict adherence to the national malaria guidelines as 44% [26]. This is common in developing countries where evidence-based interventions are available, but not enough of them have been integrated into routine practice to improve the health care system. As such, it has become more important to measure implementation outcomes and not just programme outcomes.

Fidelity of implementation is one of eight implementation outcomes. It is the degree to which a programme or protocol is implemented as intended by those who originally developed or designed it [27, 28]. Fidelity is usually measured in terms of adherence to protocols or guidelines, the amount of the intervention/programme delivered, or the quality of delivery of the intervention/programme [27]. Higher levels of adherence are usually associated with better programme outcomes [29]. Therefore, poor adherence to malaria guidelines in Nigeria is a major barrier to the achievement of the national goal of malaria elimination. Some factors that have been identified as contributors to poor adherence to malaria guidelines include, staff cadre [20, 24, 30], type of health care facility [24, 26, 30], availability of a malaria diagnostic test in the health care facility [20, 31], and availability of guidelines in the health care facility [31, 32].

Apart from contributing to drug resistance, presumptive diagnosis wastes money and time, as it results in unnecessary drug purchases and delays treatment of the true cause of febrile illness if not due to malaria [11, 26]. Rivers State, the site for this study, has seen a decline in malaria diagnosis and testing in children under the age of five years (from 19.3% in 2013 to 8.7% in 2018), with an increase in treatment of febrile illnesses with anti-malarials over the same period (from 19.7% in 2013 to 32% in 2018) [23, 33]. This decline in testing and increase in treatment with anti-malarials suggests increasing presumptive diagnosis and overtreatment with anti-malarials. There is paucity of information on adherence to the ‘test and treat” strategy in the informal private sector, particularly the proprietary patent medicine vendors (PPMVs), which experience the highest patronage of febrile patients [34]. In addition, there is still insufficient information on compliance to malaria guidelines in Nigeria using implementation frameworks. The primary objective of this study was to assess the fidelity of implementation of the national guidelines on malaria diagnosis for children under-five years in health care facilities in Rivers State, Nigeria. Secondary objectives were to describe factors that affect fidelity in this context, and to examine the associations between the moderating factors and level of fidelity of implementation of the national guidelines.

## Methods

### Study design

**Fig. 1:**
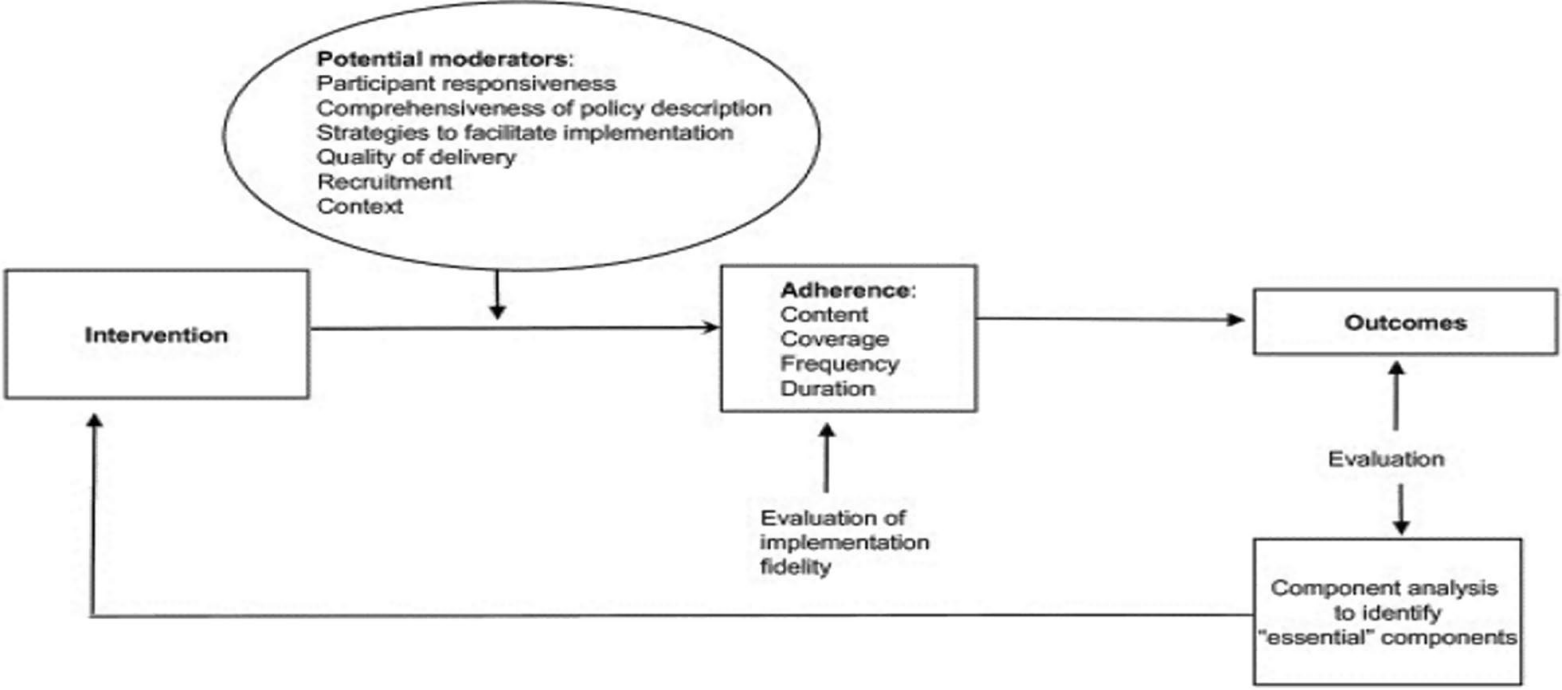
Conceptual Framework for Implementation Fidelity (after Carroll *et a*l.) [27]

This was a descriptive, cross-sectional study with analytical components. To evaluate fidelity of implementation, the Conceptual Framework for Implementation Fidelity was adapted for use as it makes adherence the baseline measurement for fidelity as seen in Fig. 1 [27]. The framework has four constructs to measure fidelity: content, coverage, frequency and duration. Since the malaria “test and treat” strategy is not delivered as a time-bound intervention, duration was not measured in this study. Potential moderators of adherence chosen from the framework include facilitation strategies, intervention complexity and participant responsiveness. The definitions of fidelity, its constructs and potential moderators defined in the context of this study are summarized in Table 1.

**Table 1:** Definitions of fidelity, its constructs and moderating factors based on the national malaria guidelines

Terms	Definitions
Fidelity	This describes how well the health care facilities followed the guidelines as intended by those who developed it. It has four constructs, three of which were applicable in this study
Constructs of fidelity	
Content	The essential or core components of the “test and treat” protocol: i. Parasitological diagnosis (testing) should be carried out for all suspected cases of malaria ii. Light microscopy and malaria rapid diagnostic tests are the two recommended methods for parasitological diagnosis iii. Anti-malarial treatment should only be initiated for patients whose diagnostic tests are positive
Coverage	How many suspected malaria cases were tested before treatment at the facility, using the last ten children aged under five years
Frequency	How often the facility performed/requested malaria diagnostic tests before treatment of children aged under five years
Moderating factors of implementation fidelity	
Facilitation strategies	Supportive measures made available to facilities by government and/or its partners to enhance the implementation of the guideline. These include training, provision of guidelines, supportive supervision, on-the-job mentoring, provision of diagnostic supplies, and others
Intervention complexity	The simplicity or complexity of the guidelines as perceived by the health care providers at the facilities
Participant responsiveness	The level of response, engagement or ownership of the guidelines by the implementers or recipients In this study, participant responsiveness was targeted at the implementers and not the patients/recipients of the “test and treat” protocol

### The “test and treat” protocol

The core components of the “test and treat” protocol based on the guidelines were defined in this study as, (i) parasitological diagnosis (testing) should be carried out for all suspected cases of malaria in children, (ii) light microscopy and malaria rapid diagnostic tests are the two recommended methods for parasitological diagnosis, and (iii) anti-malarial treatment should only be initiated for patients whose diagnostic tests are positive.

### Study setting

The study was conducted in Port Harcourt metropolis, which is made up of two local government areas, Port Harcourt and Obio/Akpor, in Rivers State. The climate is conducive for breeding of mosquitoes with annual average rainfall of 200.45 mm, average ambient temperatures between 22 °C and 31 °C, and humidity ranging between 69% and 122% [35]. It has a robust health care system consisting of public and private health care facilities at primary, secondary and tertiary level, coordinated by the State Ministry of Health.

### Sample size

The required sample size for this study was calculated using Stata version 15 (Stata Corporation, College Station, TX, USA) [36]. The mean and standard deviation of the adherence scale to be developed were not yet known at the time of sample size calculation, as there were no previous studies that have measured implementation fidelity of health facilities to national malaria guidelines using a quantitative scale. Therefore, sample size and power considerations were given for a binary variable (good fidelity vs poor fidelity). Assuming that approximately 50% of facilities had good fidelity, a sample size of 150 would allow the actual proportion to be estimated with a precision of ± 8%. The sample size calculation did not take into account the effect of clustering of health care facilities within wards as only a small number, i.e., six facilities, were selected from each ward. Further, a moderating factor, intervention complexity, was considered to be binary (complex vs non-complex), and it was assumed that 60% of respondents viewed the guidelines as “complex” and 40% viewed the guidelines as “not complex”, so the study had over 80% power to detect an absolute difference of 25% in the proportion with good fidelity between those who viewed the guidelines as “complex” and those who viewed the guidelines as “not complex”.

### Sampling design

A multi-stage sampling design was used for this study to select the 150 health facilities required for the study: 120 private and 30 public. For the public health care facilities, a sampling frame consisting of all government-operated facilities in both LGAs was obtained from the Rivers State Ministry of Health, and the Rivers State Primary Health Care Management Board. Thirty facilities were then selected through simple random sampling using a random number table. Two-stage sampling, random and systematic, were used to select private facilities as there was no comprehensive list of these facilities available. The already existing political ward structure in the State was used to randomly select twenty wards to represent approximately half of the study area (Obio/Akpor has 17 wards, while Port Harcourt has 20 wards). Six private facilities (three formal and three informal) were then selected from each ward through systematic sampling to get a total of 120 private health facilities.

### Study population

The study population comprised public and private health care facilities in the study area that provided malaria treatment services to children under five years of age. Public health care facilities were considered as those managed and operated by government at any level, while private health care facilities were defined as facilities operated by any entity besides the government—whether individuals, organizations, or religious bodies. Private health care facilities were further classified as “formal”, i.e., hospitals or clinics and drug retail outlets operated by trained pharmacists, or “informal”, i.e., PPMVs (private drug retail outlets operated by any person without formal training in pharmacy). Health facilities were included in the study if they provided malaria treatment services to children under-five years of age and had been in operation for at least six months at the time of data collection. Facilities that met the inclusion criteria but were not open for services at the time of data collection were excluded.

### Data collection

Data were collected in March 2020 by the lead author and trained data collectors using pre-tested, interviewer-administered questionnaires. The questionnaire was developed using Carroll’s Conceptual Framework for Implementation Fidelity [27]. It was divided into four sections that collected information on characteristics of the facility, characteristics of the respondent, the constructs for implementation fidelity and moderating factors. The descriptions of these constructs are detailed in Table 1. Explanatory variables for fidelity were measured as the moderating factors of fidelity i.e., facilitation strategies, intervention complexity, and participant responsiveness.

In addition, facility and respondent characteristics were collected as explanatory variables. Facility characteristics comprised of the type of facility (public, private formal or private informal), type of malaria test offered (no test, RDT and/or microscopy as the two recommended methods in the guidelines), and availability of the national malaria guidelines in the facility (yes or no). Respondent characteristics were age, sex and professional cadre. Age was categorized in ascending order, from 18 years and above, as an ordinal variable. Sex was categorized as a binary variable, either male or female, and professional cadre was categorized as a nominal variable, viz. doctor, nurse, pharmacist, community health care worker, technician and non-health care worker.

### Data analysis

Statistical analyses were conducted using Stata version 15 (Stata Corporation, College Station, TX, USA) [36]. Descriptive statistics, frequency (proportion), mean (standard deviation (SD)) and median (interquartile range (IQR)), were used to examine fidelity, facility and respondent characteristics, and the moderating factors of fidelity (some of the potential moderators of adherence from the framework chosen for this study). Inferential statistics were performed using univariable and multivariable analyses.

The outcome variable, implementation fidelity, was measured as a continuous, quantitative variable, which gave us more power to detect significant differences in the analysis. Implementation fidelity is a latent variable, i.e., it cannot be directly observed, but was derived from the three constructs: content, coverage, and frequency. These constructs were all treated as quantitative variables and the responses under each were assigned scores. Content was scored from 0 to 4, Coverage was scored from 0 to 10 and Frequency was measured on a Likert scale of 1–5. To standardize the scoring for each construct, they were assigned equal weights as the chosen conceptual framework gives no preference for one construct over the other. The weighted scores were summed up to create a percentage fidelity score with a range of 0–100%. Converting to a percentage score for fidelity was chosen for easy interpretation and comparison with prior studies.

This was created with the formula,

$${ }\frac{{\text{X}}}{{\text{Y}}}\,\,{\text{x}}\,\frac{{{100}}}{{3}}$$

Where *X* is the observed construct score, *Y* is the maximum construct score, and 100/3 is the constant weight.

The scale reliability coefficient (Cronbach’s alpha) was used to assess if the eight items on the questionnaire provided a reliable measure of implementation fidelity. Cronbach’s alpha coefficient was 0.81 which is acceptable [37].

A Shapiro–Wilk test showed fidelity to be non-normally distributed (W = 0.96, p < 0.001), therefore, Mann–Whitney U and Kruskal–Wallis tests were conducted to examine if there was a statistically significant difference between the fidelity scores of the facility and respondent characteristics. Also, the median with the interquartile range were used to summarize fidelity.

Simple linear regression was carried out for univariable analysis to investigate the relationship between each factor and fidelity score. Multiple linear regression modelling using robust estimation of errors was used for multivariable analysis to determine the relationship between the moderating factors and fidelity score. Robust estimation of standard errors was used to deal with the non-normality of fidelity score in both regression models.

The relationships are described by this linear equation:

$${\text{y}}_{{\text{i}}} \, = \,\beta_{0} \, + \,\beta_{{1}} {\text{x}}_{{{\text{i1}}}} \, + \,\beta_{{2}} {\text{x}}_{{{\text{i2}}}} \, + \, \ldots \, + \,\beta_{{\text{n}}} {\text{x}}_{{{\text{in}}}} \, + \,\varepsilon_{{\text{i}}}$$

where, y_i_ is the dependent variable, β_0_ is the intercept, x_i_ are the explanatory variables (from 1 to n), β are the coefficients of the variables (from 1 to n), and ε_i_ is the error term. Statistical significance level was set at p < 0.05.

Two multiple linear regression models were fitted. In the first model (model 1), the three moderating factors (key determinants) of implementation fidelity were fitted in the model with fidelity score. Model 2 had all factors in model 1 including facility and respondent characteristics. The proportion of variability in fidelity score explained by the fitted models (R-squared) was used to determine goodness of fit between both models.

## Results

### Health facility and respondent characteristics

Of 150 facilities administered questionnaires, a total of 147 health care facilities were included in the analysis as three facilities were discovered to have been in operation for less than six months and were excluded. Only 13% of sampled facilities had a copy of the national malaria guidelines available in the facility (Table 2). Most of the sampled health care facilities offered one of the two recommended malaria diagnostic tests, with about 25% offering RDT. About 65% of respondents were female and most were nurses (41%). Most respondents fell within the 25–34 years (29%) and 35–44 years (42%) age groups.

**Table 2:** Descriptive statistics of surveyed health facilities by facility type

Characteristics	Public	Formal private	Informal private	Total
	n (%)	n (%)	n (%)	n (%)
Age of respondent (years)				
18–24	0	7 (12.3)	4 (6.7)	11 (7.5)
25–34	5 (16.7)	22 (38.6)	16 (26.6)	43 (29.3)
35–44	20 (66.7)	16 (28.1)	27 (45)	63 (42.8)
45–54	4 (13.3)	11 (19.3)	10 (16.7)	25 (17.0)
> 54	1 (3.3)	1 (1.7)	3 (5)	5 (3.4)
Missing = 0				
Sex				
Male	10 (33.3)	25 (44.6)	16 (26.7)	51 (34.9)
Female	20 (66.7)	31 (55.4)	44 (73.3)	95 (65.1)
Missing = 1				
Cadre of respondent				
Doctor	23 (76.7)	20 (35.1)	0	43 (29.5)
Nurse	3 (10)	17 (29.8)	40 (67.8)	60 (41.1)
Pharmacist	1 (3.3)	16 (28.1)	1 (1.7)	18 (12.3)
Pharm./Lab. technician	3 (10)	0	8 (13.5)	11 (7.5)
CHEW	0	1 (1.8)	3 (5.1)	4 (2.7)
Non-health care worker	0	3 (5.2)	7 (11.9)	10 (6.9)
Missing = 1				
Type of malaria test conducted at facility				
None	0	11 (19.3)	46 (79.3)	57 (39.3)
Light microscopy	2 (6.6)	14 (24.6)	5 (8.6)	21 (14.5)
RDT	17 (56.7)	12 (21)	7 (12.1)	36 (24.8)
Both microscopy and RDT	11 (36.7)	20 (35.1)	0	31 (21.4)
Missing = 2				
Availability of national malaria guidelines				
Yes	7 (23.3)	12 (21.8)	0	19 (13.1)
No	23 (76.7)	43 (78.2)	60 (100)	126 (86.9)
Missing = 2				

### Description of moderating factors: Intervention complexity, participant responsiveness, and facilitation strategies

The scores for the three moderating factors were derived by summing item scores on the questionnaire. The mean (SD) intervention complexity score was 3 (2) out of a possible maximum score of 10. The mean (SD) participant responsiveness score was 5 (1) out of a possible maximum score of 6 (Table 3).

**Table 3:** Descriptive statistics of moderating factors of implementation fidelity

Moderating factor	Frequency	Range	Mean (SD)
Intervention complexity	147	1–10	3 (2)
Participant responsiveness	147	0–6	5 (1)

**Fig. 2:**
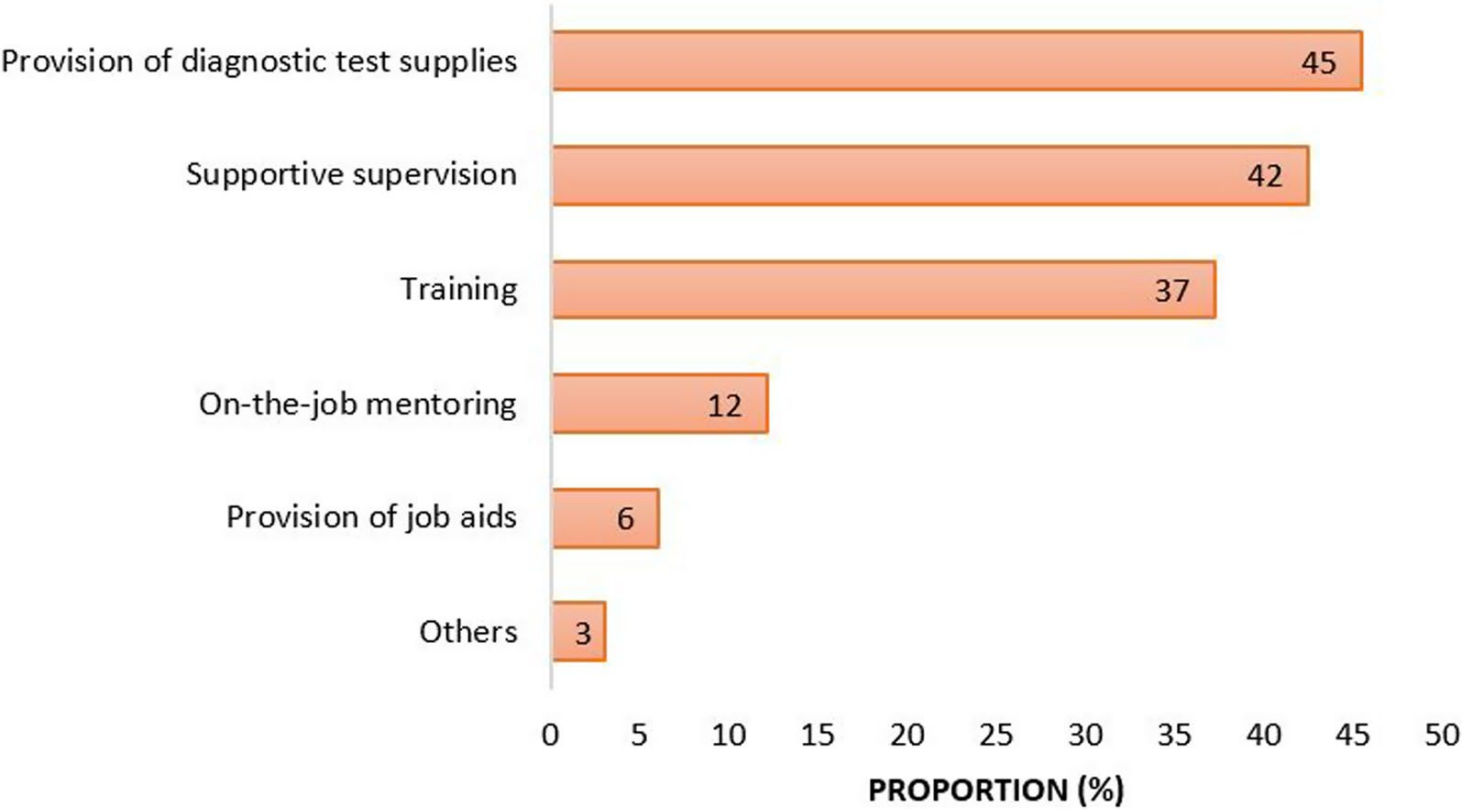
Types of support received by facilities towards implementation of the national malaria guidelines

Of 147 health care facilities, only 32 (22.5%) had received some kind of support strategy towards the implementation of the national malaria guidelines. This was mainly through the provision of diagnostic test supplies as seen in Fig. 2.

### Composite fidelity score

**Fig. 3:**
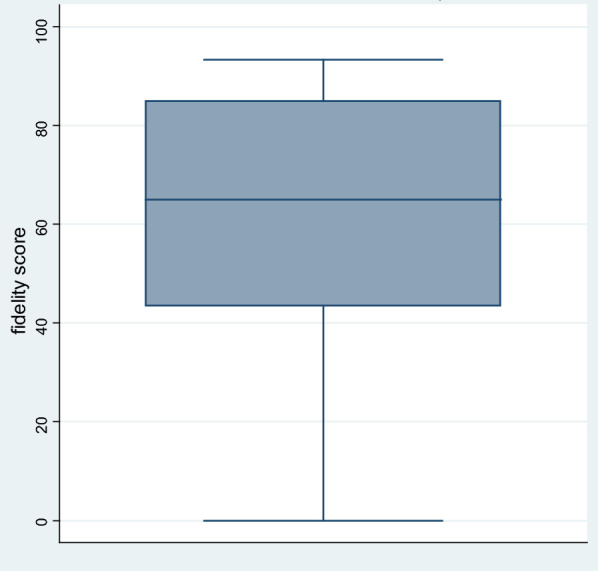
Box plot of implementation fidelity score

The minimum fidelity score was 0 and the maximum was 93.3%. The mean (SD) fidelity score for all participants was 61.3% (23.4), while the median (IQR) score was 65% (43.3, 85). The box plot in Fig. 3 shows the distribution of scores.

### Factors associated with fidelity of implementation of national guidelines for malaria diagnosis

Public health facilities had the highest median fidelity scores, same as facilities that offered both microscopy and RDT malaria diagnostic tests, and those that had the malaria guidelines available (83.3%). Differences in the median fidelity scores of all facility and respondent characteristics were statistically significant, except for age (Table 4). The median fidelity scores of facilities that had the guidelines available was almost 1.5 times higher than those facilities that did not have the guidelines (83.3% versus 59.2%, *p* < 0.001).

**Table 4:** Implementation fidelity score by facility and respondent characteristics using non-parametric tests

Variable	Fidelity score	p-value
	Median (IQR)	
Facility type		< 0.001*^a^
Public	83.3 (71.7, 85)	
Formal private	75 (51.7, 85)	
Informal private	45 (35, 60)	
Type of malaria test conducted at facility		< 0.001*^a^
None	45 (33.3, 53.3)	
Light microscopy	68.3 (53.3, 93.3)	
RDT	80 (64.2, 85)	
Both microscopy and RDT	83.33 (71.7, 90)	
Availability of national malaria guidelines		< 0.001*^b^
Yes	83.3 (71.7, 85)	
No	59.2 (40, 80)	
Age of respondent		0.125^a^
18–24	51.7 (28.3, 63.3)	
25–34	58.3 (35, 83.3)	
35–44	68.3 (46.7, 85)	
45–54	66.7 (46.7, 85)	
> 54	73.3 (71.7, 76.7)	
Sex		0.005*^b^
Male	71.7 (48.3, 85)	
Female	60 (38.3, 80)	
Cadre of respondent		< 0.001*^a^
Doctor	85 (71.7, 85)	
Nurse	46.67 (35.8, 65)	
Pharmacist	60.83 (50, 80)	
Pharmacy or laboratory technician	64.17 (28.3, 93.3)	
Community health extension worker	48.33 (40, 63.3)	
Non-health care worker	55 (45, 85)	

^*^Significant at p < 0.05; ^a^ Kruskal–Wallis test; ^b^Mann-Whitney U test

Unadjusted estimates with simple linear regression modelling for the key moderating factors, facility and respondent characteristics were all significant at the 5% level (Additional file 1: Table S1). For multiple regression analysis, the full model (model 2) with all the determinants accounted for about 66% of the variability in the outcome, considering the proportion of variability in fidelity score explained by the fitted models (Table 5). Therefore, it can be said that model 2 is a better predictor of fidelity score in the context of this study and will be used to present the regression results. Intervention complexity had a statistically significant inverse relationship to implementation fidelity (β = − 1.89, 95% CI [− 3.42, − 0.34], p = 0.017). Participant responsiveness was positively and significantly associated with fidelity score (β = 8.6, 95% CI [4.8, 12.3], p < 0.001). Facilitation strategies was only marginally significant in the final model (β = 5.7, 95% CI [− 0.31, 11.76], p = 0.063). Type of malaria test conducted at the facility was the only other factor that was significant at the 5% level.

**Table 5:** Multiple linear regression showing association between moderating factors only, and all factors

Factor	Model 1		Model 2	
	Coefficient (95% CI)	P-value	Coefficient (95% CI)	P-value
Moderating factors				
Intervention complexity	− 0.24 (− 1.73, 1.25)	0.752	− 1.88 (− 3.42, − 0.34)	0.017*
Participant responsiveness	10.56 (5.08, 16.03)	< 0.001*	8.57 (4.83, 12.32)	< 0.001*
Facilitation strategies				
No	ref	< 0.001*	ref	0.063
Yes	19.27 (12.71, 25.83)		5.73 (− 0.31, 11.76)	
Facility type				0.171
Public			ref	
Formal private			− 6.10 (− 12.79, 0.58)	
Informal private			− 7.40 (− 17.57, 2.76)	
Type of malaria test conducted at facility				< 0.001*
None			ref	
RDT			16.90 (6.78, 27.03)	
Light microscopy			21.88 (13.60, 30.16)	
Both microscopy and RDT			26.63 (16.88, 36.38)	
Availability of national malaria guidelines				0.176
No			ref	
Yes			6.14 (− 2.80, 15.09)	
Duration of facility operation			0.01 (− 0.02, 0.04)	0.631
Age of respondent			3.19 (− 0.42, 6.80)	0.082
Sex				0.722
Male			ref	
Female			1.09 (− 4.96, 7.14)	
Cadre of respondent				0.075
Doctor			ref	
Nurse			− 7.50 (− 16.38, 1.38)	
Pharmacist			− 6.26 (− 15.98, 3.45)	
Pharmacy or laboratory technician			− 5.94 − 15.51, 3.63)	
Community health extension worker			8.74 (− 10.55, 28.02)	
Non-health care worker			5.72 (− 7.45, 18.90)	
Model R-squared^a^	31%		65.7%	
Model p-value	< 0.001		< 0.001	

^*^significant at p < 0.05

^a^R-squared—the proportion of variance of fidelity score explained by the factors in the model

## Discussion

This study found fidelity of implementation of the national guidelines on malaria diagnosis to be moderate (using 50% as the minimum acceptable standard on a percentage scale). The main model showed that the factors significantly associated with implementation fidelity were intervention complexity, participant responsiveness, and the type of malaria test conducted at the facility. There were significant differences in the median fidelity scores of all health facility characteristics, with public health facilities, facilities that offered both microscopy and RDT malaria diagnostic tests, and those that had the malaria guidelines available having the highest median fidelity scores compared to their counterparts. The median fidelity score for facilities that participated in this study is similar to that reported in a health care facility survey in Papua New Guinea where overall adherence to the malaria “test and treat” protocol was 63% [21]. It is higher than adherence scores from other studies conducted in Nigeria that sampled both public and private facilities and reported adherence scores below 50% [24–26, 38, 39]. However, it implies that core components of the malaria “test and treat” strategy are not still being implemented as intended at these facilities.

There was an inverse relationship between intervention complexity and implementation fidelity. This indicates that the more complex the users thought the guidelines were, the lower their adherence to it, and vice versa. Increase in participant responsiveness score was positively and significantly associated with increase in fidelity score suggesting that a higher acceptance of the guidelines by facility respondents led to better adherence. These are consistent with findings from other studies that have investigated the potential moderators of fidelity to health care interventions [40, 41]. While there was some evidence to show that facilitation strategies were associated with implementation fidelity, it was not significant in the final model. This is quite different from other studies on malaria guidelines that reported strategies like training and provision of job aids were associated with higher levels of adherence to protocols [20, 31, 42]. However, it has been said that facilitation strategies do not necessarily translate into better implementation fidelity [40]. It is usually dependent on other moderators of the intervention, for example, highly motivated staff will likely implement with high fidelity even with limited facilitation [40].

Type of malaria test offered at health facilities also had very strong association with fidelity score in the regression model. From the univariable analysis, there was significant difference in the median fidelity scores of health facilities based on the types of malaria test they offered. Health care facilities that offered both light microscopy and RDT tests had the highest median fidelity scores, compared with those that offered one type of test or none at all. A similar result was reported in a survey in Kenya monitoring implementation of the malaria “test and treat” policy [31]. Therefore, it is important for facilities offering malaria treatment to children to offer at least one of the diagnostic tests, for instance RDTs, which require little skill and are recommended for use in almost all settings.

### Study limitations

The findings from this study will not be generalizable to other contexts as it is a cross-sectional study and will only be applicable to health care facilities in Port Harcourt metropolis, Rivers State. However, since there has been no other study in the State that has assessed fidelity of implementation to the malaria “test and treat” strategy in public, formal private and informal private facilities, it will contribute to the knowledge base for academic purposes and policy making in similar settings. Social desirability bias was a possibility as the study participants could have given responses to appear favourable or knowledgeable, which is common with most self-reported evaluations. To mitigate this, the questionnaire was pretested, and questions structured in the most suitable way to capture the true responses. It is also possible that other potential confounders of fidelity may exist outside those assessed which the study was not able to control for as it is a cross-sectional study. The absence of a standardized tool for measuring implementation fidelity to malaria intervention programmes also affected the development of the data collection tool, which had to be created without reference to any existing parameters. However, measuring adherence based on a quantitative percentage scale, as was done in this study, can provide a template for the development of similar tools.

## Conclusions

This study was able to measure the fidelity of implementation of health care facilities in Rivers State to the “test and treat” strategy of the national malaria guidelines, when managing children under the age of five years. Adherence to the malaria “test and treat” strategy is well below 100% suggesting presumptive diagnosis is still widely practiced, especially in informal private health facilities which had the lowest fidelity score. The fact that facilities are not implementing the guidelines at high fidelity is a major barrier to the achievement of the goals of the National Malaria Strategic Plan, one of which is to ensure that all suspected cases are tested for malaria before treatment. Intervention complexity, participant responsiveness and the type of malaria test conducted at the facility were identified as moderating and contextual factors that can explain the level of adherence. It has lent credence to the fact that policies and interventions should be perceived as simple not complex, by implementers, for better adherence. Also, further education of health care providers on the relative advantage of the “test and treat” strategy over presumptive diagnosis might improve programme ownership, and thus, increase adherence. The significant association between parasitological diagnostic tests and implementation fidelity should encourage policy makers and implementing partners to continue supporting facilities with diagnostic test supplies. Priority should be placed on training and re-training on the use of RDTs especially in the private health care sector, with an emphasis on PPMVs. In the same vein, the most current edition of the national malaria guidelines should be made available to all health care facilities that manage cases of malaria, and where available, it should be easily accessible to all service providers in the facility. Given the proliferation of interventions in Africa with paucity of data on measuring implementation outcomes, there is need for further empirical research on how implementation fidelity can be measured in the local context, using conceptual frameworks.

### Supplementary Information

**Additional file 1: Table S1.** Associations between facility and respondent characteristics, and implementation fidelity score using univariable linear regression analysis.

## Data Availability

The dataset analysed during this study may be available upon reasonable request made to the corresponding author.

## Abbreviations

LGA: Local government area
PPMV: Proprietary patent medicine vendor
RDT: Rapid diagnostic test
WHO: World Health Organization
