# Supplementary material for: Fidelity of implementation of national guidelines on malaria diagnosis for children under-five years in Rivers State, Nigeria
Source: Malar J. 2024 Apr 27;23:123. doi: 10.1186/s12936-024-04957-4 (PMC11055277; doi:10.1186/s12936-024-04957-4)
Supplement: Supplementary file 1 — Additional file 1: Table S1. Associations between facility and respondent characteristics, and implementation fidelity score using univariable linear regression analysis. [file 12936_2024_4957_MOESM1_ESM.docx]

**Fidelity of implementation of national guidelines on malaria diagnosis for children under-five years in Rivers State, Nigeria**

Whyte Mina^1,2*,^ Slemming Wiedaad^3,4^, Levin Jonathan^1^

^1^Division of Epidemiology and Biostatistics, School of Public Health, University of the Witwatersrand, South Africa

^2^Department of Medicine, University of Otago, Wellington, New Zealand

^3^Department of Paediatrics and Child Health, University of the Witwatersrand, South Africa

^4^Children’s Institute, University of Cape Town, Cape Town, South Africa

*Corresponding author: Mina Whyte, whymi085@student.otago.ac.nz

**Supplemental Table**

Table S1. Associations between facility and respondent characteristics, and implementation fidelity score using univariable linear regression analysis

| **Variable** | **Coefficient (95% C.I.)** | **P-value** |
| --- | --- | --- |
| **Moderating factor** | | |
| Intervention complexity | 1.85 (0.52, 3.19) | 0.007* |
| Participant responsiveness | 11.07 (5.54, 16.60) | <0.001* |
| Facilitation strategies  No  Yes | ref  21.39 (15.07, 27.72) | ref  <0.001* |
| **Facility type** | | <0.001* |
| Public | ref |  |
| Formal private | -11.95 (-18.97, -4.93) |  |
| Informal private | -32.39 (-39.20, -25.59) |  |
| **Type of malaria test conducted at facility** | | <0.001* |
| None | ref |  |
| Light microscopy | 27.22 (17.93) |  |
| RDT | 29.35 (21.60, 37.10) |  |
| Both microscopy and RDT | 35.56 (28.32, 42.80) |  |
| **Availability of national malaria guidelines** | | <0.001* |
| No | ref |  |
| Yes | 20 (54.32, 62.68) |  |
| **Duration of facility operation** | 0.07 (0.04, 0.11) | <0.001* |
| **Age of respondent** | 5.68 (2.12, 9.23) | 0.002* |

*Significant at p < 0.05

| **Variable** | **Coefficient (95% C.I.)** | **P-value** |
| --- | --- | --- |
| **Sex** | | 0.005* |
| Male | ref |  |
| Female | -10.98 (-18.56, -3.41) |  |
| **Cadre of respondent** | | <0.001* |
| Doctor | ref |  |
| Nurse | -30.00 (-36.76, -23.23) |  |
| Pharmacist | -16.46 (-26.57, -6.35) |  |
| CHEW | -29.13 (-40.44, -17.81) |  |
| Pharm./Lab. Technician | -19.05 (-52.37, 14.27) |  |
| Non-health care worker | -19.05 (-32.95, -5.15) |  |
